# Dietary fructose and high salt in young male Sprague Dawley rats induces salt‐sensitive changes in renal function in later life

**DOI:** 10.14814/phy2.15456

**Published:** 2022-09-19

**Authors:** Peter E. Levanovich, Ana M. Daugherty, Dragana Komnenov, Noreen F. Rossi

**Affiliations:** ^1^ Department of Physiology Wayne State University Detroit Michigan USA; ^2^ Department of Psychology and Institute of Gerontology Wayne State University Detroit Michigan USA; ^3^ Department of Internal Medicine Wayne State University Detroit Michigan USA; ^4^ John D. Dingell VA Medical Center Detroit Michigan USA

**Keywords:** critical development period, fructose, glomerular filtration rate, salt sensitive blood pressure

## Abstract

Dietary fructose and salt are associated with hypertension and renal disease. Dietary input during critical postnatal periods may impact pathophysiology in maturity. The highest consumption of fructose occurs during adolescence. We hypothesized that a diet high in fructose with or without high salt in young male Sprague Dawley rats will lead to salt‐sensitive hypertension, albuminuria, and decreased renal function in maturity. Four groups were studied from age 5 weeks: 20% glucose + 0.4% salt (GCS‐GCS) or 20% fructose + 4% salt throughout (FHS‐FHS). Two groups received 20% fructose + 0.4% salt or 20% fructose + 4% salt for 3 weeks (Phase I) followed by 20% glucose + 0.4% salt (Phase II). In Phase III (age 13–15 weeks), these two groups were challenged with 20% glucose + 4% salt, (FCS‐GHS) and (FHS‐GHS), respectively. Each group fed fructose in Phase I exhibited significantly higher MAP than GCS‐GCS in Phase III. Net sodium balance, unadjusted, or adjusted for caloric intake and urine flow rate, and cumulative sodium balance were positive in FHS during Phase I and were significantly higher in FCS‐GHS, FHS‐GHS, and FHS‐FHS vs GCS‐GCS during Phase III. All three groups fed fructose during Phase I displayed significantly elevated albuminuria. GFR was significantly lower in FHS‐FHS vs GCS‐GCS at maturity. Qualitative histology showed mesangial expansion and hypercellularity in FHS‐FHS rats. Thus, fructose ingestion during a critical period in rats, analogous to human preadolescence and adolescence, results in salt‐sensitive hypertension and albuminuria in maturity. Prolonged dietary fructose and salt ingestion lead to a decline in renal function with evidence suggestive of mesangial hypercellularity.

## Graphical abstract

Dietary fructose eaten in young rats at levels comparable to the upper quintile of humans results in positive sodium balance and salt sensitive blood pressure when placed on a high salt diet in maturity. Fructose is eaten together with high salt in youth results in albuminuria and lower glomerular filtration rate in adult rats.

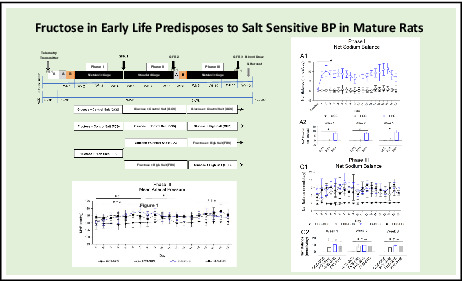

## 1 INTRODUCTION

According to the American College of Cardiology and American Heart Association 2017 Clinical Practice Guidelines (Whelton et al., 2018a; Whelton et al., 2018b), 47.3% of the U.S. population is hypertensive with the majority not well controlled (Ostchega et al., 2020). Environmental and lifestyle factors such as diet have become important contributors to hypertension. Ingestion of fructose‐sweetened beverages and food products (Bray et al., 2004; Jayalath et al., 2015; Welsh et al., 2005) along with high sodium intake (Brouillard et al., 2020; Hu et al., 2020) has persisted in both adolescents and adults despite efforts to improve dietary habits. Dietary fructose and sodium ingestion have been directly correlated to cardiovascular and renal diseases (Farquhar et al., 2015; Kalogeropoulos et al., 2015). A diet high in fructose induces a state of increased sodium retention that leads to hypertension well before the development of metabolic syndrome or frank diabetes mellitus (Gordish et al., 2017; Komnenov et al., 2020; Levanovich et al., 2021). Adolescents and young adults, particularly individuals from lower income levels, are the group consuming the highest amounts of fructose, which is sourced predominately through sugar‐sweetened beverages (Bray et al., 2004). The Coronary Artery Risk Development in Young Adults (CARDIA) cohort (*n* = 240,508) (Jayalath et al., 2015) which is cited by the American Heart Association update on stroke reports a 12% greater risk of hypertension with consumption of sugar‐sweetened beverages when controlled for sex, age, race, BMI, and smoking behaviors (Benjamin et al., 2019). Despite these findings, there are few studies investigating the chronic effects of high fructose either with or without high salt intake in youth, and the potential impact later in life.

Although monogenic hypertension syndromes exist (Levanovich et al., 2020), hypertension is largely polygenic and influenced by epigenetic factors (Padmanabhan et al., 2017). Full phenotypic manifestation does not generally occur before adulthood. The insults that shape disease progression and extent can often be traced back to early periods of development, or ontogeny (Barker, 1995). The development of organs and physiologic systems can be attributed to specific time points; chiefly, the earliest developmental stages of these systems are marked by the most rapid growth. During these windows of development, the application of external stimuli can exert influence that alters physiologic function. These time frames are referred to as critical developmental periods. In rats, readjustment of central hemodynamic function in response to postnatal stimuli can occur during prepuberty and sexual maturation periods, largely between 4 and 6 weeks after birth (Albrecht, 1974; Folkow & Svanborg, 1993). Although programming is known to take place during morphogenesis and fetal development, maturation or realignment of blood pressure governing systems such as the baroreflex and renin‐angiotensin‐aldosterone system (RAS) may occur during postnatal critical periods (Andresen et al., 1980; Jelinek et al., 1990; Pohlova & Jelinek, 1974; Su et al., 1992).

In several preclinical models, a high salt diet in early life induces salt‐sensitive hypertension that may not necessarily occur when the same challenge is applied in adulthood (Brownie et al., 1966; Kunes & Jelinek, 1984; Musilova et al., 1966; Zicha & Kunes, 1994). Likewise, various transient antihypertensive therapies applied for a limited time during early life attenuate the development and degree of hypertension in genetically predisposed strains such as the spontaneously hypertensive rat (Adams et al., 1990; Benetos et al., 1994; Weiss & Lundgren, 1978). Notably, this effect coincides with the same critical time periods (4 to 9 weeks of age) used in salt sensitivity programming via excess sodium loading, further emphasizing the importance of these critical time periods in hypertension induction.

The impact of fructose programming on neonates and adolescents is less well understood. Glucose transporter 5 (Glut5) transports fructose and is considered critical to the development of fructose‐induced salt‐sensitive hypertension (Singh et al., 2008). Glut5 is underexpressed in neonatal intestinal tissue. After weaning, there is an abrupt increase in Glut5 mRNA and protein expression which is enhanced if fructose is provided during weaning (Jiang & Ferraris, 2001; Toloza & Diamond, 1992). Emerging data show that maternal intake of high fructose results in epigenetic changes associated with hypertension later in life (Cho & Kim, 2020; Koo et al., 2021). Our group has demonstrated that high fructose combined with high salt ingested between 5 and 9 weeks of age leads to salt‐sensitive hypertension and arterial stiffness in maturity along with diastolic cardiac dysfunction in adult male rats (Levanovich et al., 2020). Little is known about the effects of fructose ingestion in young postnatal life and renal handling of sodium later in maturity. A required first step requires demonstrating whether high fructose intake during a limited period in youth will impact renal function and sodium excretion in maturity. Thus, the purpose of the present studies was to assess the impact of 20% dietary fructose (an amount comparable to that ingested by the upper quintile of the adolescent population) either alone or with 4% sodium during rat age analogous to human pre‐ and early adolescence (Sengupta, 2013; Varlinskaya & Spear, 2008) will impact blood pressure, glomerular filtration rate, and urinary excretion of sodium, potassium, and albumin. We hypothesize that a diet high in fructose, but not glucose, either with or without high salt intake in male Sprague Dawley rats aged 5–8 weeks will be sufficient to lead to salt‐sensitive hypertension, decreased renal function, and albuminuria in maturity.

## 2 MATERIALS AND METHODS

### 2.1 Animals

Since previous reports were almost exclusively performed in male rats, our studies were performed in male Sprague Dawley rats to permit assessment of the impact of fructose in the context of existing studies. The rats (Envigo Sprague Dawley) were housed under controlled conditions (21–23°C; 12‐h light/dark cycles, lighting period 06:00 to 18:00 pm). Complete care provided to rats was in accordance with the principles of the National Institutes of Health *Guide for the Care and Use of Laboratory Animals*. All procedures and protocols were approved by the Wayne State University Institutional Animal Care and Use Committee (Protocol #19‐03‐1001).

### 2.2 Dietary regimen

**FIGURE 1:**
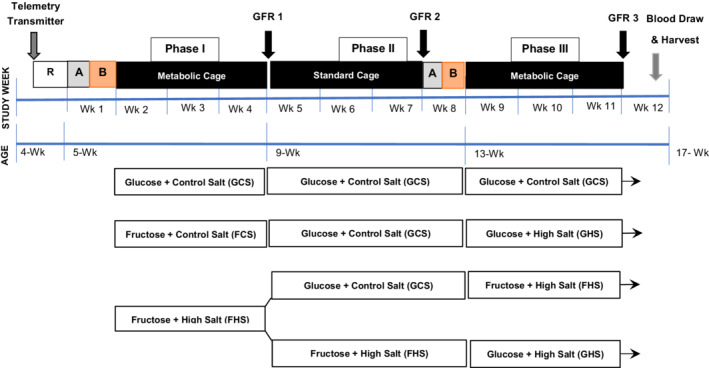
Schematic of the protocol. The age of the rats, study week, and Phases I, II, and III are shown across time. R, recovery; A, acclimation; B, baseline. Hemodynamic transmitter placement and measurement of GFR are indicated. In the text and subsequent figures, the groups are named for their fructose‐salt intake during Phases I and III, respectively, GCS‐GCS, FCS‐GHS, FHS‐GHS, and FHS‐FHS.

Upon arrival, rats were acclimated to standard polyurethane caging and were permitted ad libitum access to water and standard rat chow for a minimum of 48 hours prior to enrollment in the experimental protocols. At the age of 4–5 weeks (body weight ~ 125 g), rats underwent placement of hemodynamic telemetry transmitters and recovered in individual housing for 3 days. Following recovery, rats were placed into metabolic housing units (Tecniplast USA) equipped with custom‐fitted holders for telemetry receivers (Data Sciences Intl) and randomly assigned to diets containing either 20% fructose or 20% glucose with 0.4% sodium. These diets were provided ad libitum for 4–5 days to permit baseline hemodynamic recordings. Then, the rats were formally entered by random assignment into the groups for the protocol (Figure 1).

#### 2.2.1 Phase I (Days 0–22 ± 2)

The glucose‐fed rats continued to be fed chow containing 20% glucose and 0.4% sodium (glucose control sodium—GCS; ModTest Diet® 5755—5WZZ; *n* = 10). The fructose‐fed rats were further randomized to receive chow with 20% fructose plus 0.4% sodium (fructose control sodium—FCS; ModTest Diet® 5755‐5W3Y, *n* = 9) or 20% fructose plus 4.0% sodium (fructose high sodium—FHS; ModTest Diet® 5755‐5WZ8; *n* = 18) for 3 weeks. Rats were pair‐fed indexed to overall caloric intake to account for differences in energy profiles (kcal/g) among the dietary formulations. Water provision in all groups remained ad libitum throughout the study. Food was replenished and urine retrieved each day between 1700 and 1730, immediately before lights off.

#### 2.2.2 Phase II (Days 23–44 ± 2)

The rats were returned to singly housed, polyurethane cages in which food and water were provided ad libitum. All rats on the GCS and FCS diets and 50% of the rats on the FHS diet were placed on the GCS diet. The remainder of the FHS rats remained on the FHS diet.

#### 2.2.3 Phase III (Days 45–66 ± 2)

Rats were then returned to metabolic housing units and were permitted a 3‐day acclimation period. Rats fed fructose and 0.4% sodium in Phase I were subjected to a high salt challenge with 20% glucose and 4.0% sodium (glucose high sodium—GHS; ModTest Diet® 5755‐5WOW). As a control, the GCS group from Phase I remained on the GCS diet. This resulted in four overall groups: (1) GCS‐GCS, (2) FCS‐GHS, (3) FHS‐GHS, and (4) FHS‐FHS. This regimen was continued for an additional 3 weeks at which time measurements of glomerular filtration rate (GFR) and terminal studies were conducted.

### 2.3 Surgical procedures

#### 2.3.1 Hemodynamic telemetry transmitter placement

All surgical procedures were conducted under intraperitoneal ketamine (80 mg/kg; Mylan Institutional, LLC) and xylazine (10 mg/kg; Akorn Animal Health, Inc.) anesthesia and subcutaneous administration of buprenorphine SR (0.3 mg/kg) for analgesia. For hemodynamic telemetry unit placement, an inguinal incision was made and the right femoral artery was exposed. Permanent ligation of the distal artery was accomplished using a 3–0 silk suture while a proximal segment of the vessel was temporarily occluded using a vascular clamp. A small arterial incision was made and the gel‐filled catheter of the hemodynamic transmitter (HDS‐10, Data Sciences International, MN) was inserted into the vessel using a 21‐gauge needle as a guide. The vascular clamp was then removed and the catheter advanced into the abdominal aorta. The transmitter body was subcutaneously tunneled dorsally to the right flank, and the incision was closed using surgical staples.

#### 2.3.2 Vascular catheter placement

At the end of Phase III, catheters were placed into the left carotid artery and external jugular vein using the anesthetic and analgesic regimen above as previously performed in our laboratory (Komnenov et al., 2020; Soncrant et al., 2018). Catheters were anchored using a 3–0 silk suture, tunneled subcutaneously, and exteriorized between the scapulae. All incisions were closed using 4–0 Prolene suture (Ethicon, Johnson & Johnson). The catheters were filled with heparinized saline (1000 units/ml) and secured. The rats were then permitted to recover in individual standard cages.

### 2.4 Analytical measurements and calculations

#### 2.4.1 Telemetry

Acquisition of hemodynamic data was conducted using Ponemah software (Data Sciences International, MN). Measurement of systolic blood pressure (SBP), diastolic blood pressure (DBP), mean arterial pressure (MAP), and heart rate (HR) were sampled for 10 s every 4 min at a sampling rate of 500 samples/second. Pulse pressure (PP) was calculated separately using these values. Baseline measurements were averaged over 3 days following a 3‐day cage acclimation. Sampling was performed at this rate continuously throughout each study phase within metabolic cages.

#### 2.4.2 Metabolic assessment

Water intake, chow consumption, and urine volume were measured gravimetrically. Urinary sodium and potassium were measured via flame photometry using an internal lithium standard (Cole Parmer Instruments).

#### 2.4.3 Transcutaneous assessment of renal function

Glomerular filtration rate (GFR) was assessed at the end of each phase (Figure 1) using venous bolus injections of fluorescein–isothiocyanate–(FITC)–sinistrin as described by Schock‐Kusch et al. (2009, 2013). Rats were briefly anesthetized with 3% followed by 1% isoflurane and a small adhesive patch with an LED‐emitting optical transducer (Mannheim Pharma and Diagnostics GmbH) was placed on a shaved region on the dorsal aspect of the thorax. Baseline measurements were recorded for 3–5 min, thereafter a single bolus injection of FITC‐sinistrin, 6 mg/100 g BW (Fresenius‐Kabi) was administered via tail vein (Phases I and II) or venous catheter (Phase III). Rats recovered from the anesthesia in singly housed polyurethane cages and measurements were recorded for 2 h. FITC‐sinistrin disappearance kinetics were analyzed via a three‐compartment approach using MB Studio software (Mannheim Pharma and Diagnostics GmbH). GFR was calculated using the following formula (Schock‐Kusch et al., 2013):

$$\mathrm{GFR}\left(\mathrm{ml}/\min/100g\;\mathrm{BW}\right)=\frac{31.26\left(\mathrm{ml}/100g\;\mathrm{BW}\right)}{t_{1/2}\left(\text{FITC}-\text{sinistrin}\right)\left(\min\right)}.$$

#### 2.4.4 Terminal procedures and hormonal assessment

Following the GFR assessment after Phase III, the rats were permitted a 6‐ to 8‐h recovery period with no access to food before terminal harvest. All blood samples were obtained in conscious rats. Fasting glucose levels were determined using a One‐Touch Ultra glucose monitor (LifeScan, Inc.) on whole blood collected directly from the arterial catheter. Blood was then collected via the carotid arterial catheter into prechilled tubes for hormonal analysis. To assay plasma renin activity (PRA), 1 ml of blood was collected into a tube containing 50 μl sodium ethylenediaminetetraacetic acid (EDTA). An additional 1 ml of blood was collected into a separate tube containing 120 μl of 500 mM sodium EDTA, 125 mM phenanthroline, 1 mM phenylmethanesulfonyl fluoride, 20 mM pepstatin, 1 mM enalapril, and 10× phosphatase inhibitor cocktail for insulin assessment. Once collected, the blood was immediately centrifuged at 3000 rpm for 4 min at 4°C. Plasma was stored at −70°C until assayed.

Rats were then euthanized with sodium pentobarbital (120 mg/kg, iv). Kidneys were harvested, kept at 0°C in saline, and weighed. A segment of the renal cortex was excised and stored at −70°C. A slice of kidney tissue was placed into formalin for 48 h and paraffin embedded and stained with periodic acid‐Schiff for histology.

### 2.5 Statistical analyses

Data were analyzed with and without adjustment (see below). Between‐group differences in the pattern of change over the study design were tested in a repeated‐measure ANOVA framework (SPSS v. 27), interpreting multivariate statistics as a profile analysis of longitudinal change within‐ and between‐groups. Based on the initial visual evaluation, the trajectories of change appear to be nonlinear; therefore, hypotheses were tested for group differences in change across days separately for weeks 1–3 of each phase, adjusting for multiple comparisons with Bonferroni correction. Group was tested as a four‐level factor for all analyses using the Phase III randomized assignment, which ensured equivalent statistical power across the study design. Based on the cell size, Pillai's multivariate *F*‐statistic was interpreted as a robust omnibus test (Bonferroni adjusted *α*’ = 0.02). Omnibus effects were interpreted with data visualization and post hoc one‐way ANOVA of between‐group differences in specific days to understand the rate of change over the week period as well as pairwise *t*‐tests for between‐subject average differences. Of the available sample across all measurements, 11.5% of data were missing at random (Little's *χ* ^2^ [7629] = 1368.98, *p* = 0.99) and were replaced with the group means. Data were missing due to random technical issues such as brief (e.g., 15 min) electrical interruption of telemetry data acquisition due to building generator tests, loss of a urine specimen due to dislodging of the collection vial from the metabolic cage, and similar issues.

Net sodium was adjusted for daily caloric intake and urine volume via residualization applied to the entire sample that is similar to ANCOVA (Garcia et al., 2020; Jack Jr. et al., 1989), and by adjusting daily values separately, this approach is consistent with time‐varying covariates in the profile analysis. Prior to hypothesis testing, data were screened for univariate outliers (|*z*| > 3.29), which identified 14 animals (37%) with at least one extreme value across the study design; the Mahalanobis distance procedure for multivariate outlier detection identified no cases. ANOVA is a robust estimation procedure with nonnormally distributed data and to avoid potential bias from outlier values, analyses were repeated with listwise deletion of those cases to determine if estimated effect size was similar. With the available sample size, the test of the between‐x within‐group effect have 80%–95% power to detect moderate effect sizes (*f* = 0.22–0.25) to statistical significance (*α*’ = 0.02; average correlation among measure = 0.5).

## 3 RESULTS

Some parameters have been reported elsewhere in the context of the impact of these dietary regimens on cardiovascular function (Levanovich et al., 2021). Data immediately relevant to the present findings that are reported herein and were provided in the previous manuscript will be clearly noted as such, either in the text or in the legends. One rat in the initial FHS group required euthanasia after sustaining an injury to his foot in the metabolic cage early in Phase I and all its data were censored.

The Initial and final body weights of the rats in each group did not differ; however, the kidney weight/body weight values of the FCS‐GHS and FHS‐FHS groups were significantly greater than those of the GCS‐GCS group (Table 1). Although the kidney weight/body weight measurements of the FHS‐GHS rats were also greater than those of the GCS‐GCS rats, they did not differ significantly from those of any of the other groups.

**TABLE 1:** Body and kidney weights

Dietary regimen	*n*	Initial weight (g)	Final body weight (g)	Left kidney weight (g/kg)	Right kidney weight (g/kg)
GCS‐GCS	9	125 ± 4	380 ± 27	3.3 ± 0.3	3.2 ± 0.2
FCS‐GHS	9	132 ± 4	347 ± 30	3.7 ± 0.4*	3.5 ± 0.4*
FHS‐GHS	8	128 ± 3	362 ± 33	3.5 ± 0.3	3.4 ± 0.2
FHS‐FHS	9	127 ± 5	366 ± 36	3.7 ± 0.3*	3.7 ± 0.2*

*Note*: Values are mean ± SE. Initial and final body weights have been published earlier (Levanovich et al., 2021).

* *p* < 0.05 vs GCS‐GCS group.

### 3.1 Caloric and fluid intakes and urine outputs

All rats in metabolic cages in Phases I and III were placed on a pair‐feeding paradigm. Chow consumption was assessed daily. Since the caloric contents of the diets were not identical, the amount provided was determined by caloric consumption rather than direct gravimetric assessment. This protocol design was implemented to limit differences, as much as possible, in total nutritional consumption due to the varying energy profiles of each type of chow.

**FIGURE 2:**
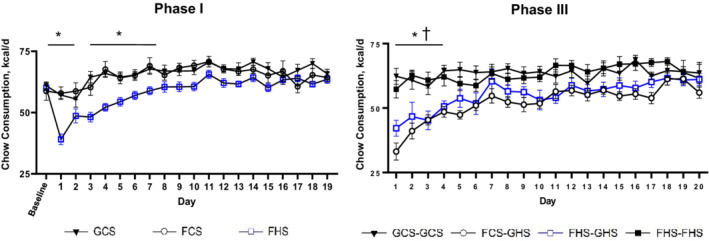
Caloric intake. Chow consumption in total kcal/d for each of the groups during Phase I and Phase III. Despite acclimation to the metabolic cages and careful attempts to pair all caloric intakes, rats consumed less chow after being switched either from baseline diet (GCS) to high salt‐containing chow (FHS) at the beginning of Phase I: *p < 0.05 FHS (*n* = 17) vs GCS (*n* = 9) or FCS (*n* = 9). Likewise, rats switched from the GCS diet during Phase II to GHS during Phase III initially consumed fewer calories: * *p* < 0.05 FCS‐GHS (*n* = 9) vs either GCS‐GCS (*n* = 9) or FHS‐FHS (*n* = 9), †*p* < 0.05 FHS‐GHS (*n* = 8) vs either GCS‐GCS or FHS‐FHS.

Table 2 depicts the average daily caloric intake, fluid intake, and urine output during each week of Phases I and III. Caloric intakes increased incrementally in each of the groups during the first 7–9 days of Phase I due to expected growth and stabilized to adult intakes by week 3 (Figure 2). Despite every effort to acclimate to the metabolic cages and to pair caloric intakes, statistical differences in caloric intake were observed in the first few days following the dietary change from baseline (0.4% sodium) to 4% salt chow in the FHS group in Phase I. Likewise, the change from GCS during Phase II to GHS for Phase III by the FCS‐GHS and FHS‐GHS groups resulted in a decline in overall caloric intake during the initial days that is reflected in week 1 of Phase III. The differences in caloric intake stabilized by the end of week 1 and through weeks 2 and 3. In contrast, the GCS‐GCS and FHS‐FHS groups that received GCS and FHS chow during Phase II, respectively, did not require adjustment to a high salt diet in Phase III and displayed a more constant day‐to‐day intake from beginning to the end of the phase (Figure 2).

**TABLE 2:** Weekly average daily caloric and fluid intakes and urinary volumes during Phase I and Phase III

Phase I										
Dietary regimen	*N*	Week 1			Week 2			Week 3		
		Caloric Intake (kcal/day)	Fluid Intake (ml/day)	Urine Output (ml/day)	Caloric Intake (kcal/day)	Fluid Intake (ml/day)	Urine Output (ml/day)	Caloric Intake (kcal/day)	Fluid Intake (ml/day)	Urine Output (ml/day)
GCS	9	61.5 ± 5	30.4 ± 8	13.7 ± 3	68.4 ± 4	33.6 ± 4	16.4 ± 3	66.0 ± 6	33.0 ± 5	15.3 ± 3
FCS	9	60.7 ± 8	32.5 ± 4	17.9 ± 3	67.2 ± 4	34.4 ± 5	19.0 ± 4	65.7 ± 5	34.3 ± 4	19.2 ± 3
FHS	17	51.9 ± 7*	73.8 ± 17*	56.2 ± 16*	63.9 ± 4	89.7 ± 16*,#	70.0 ± 17*	64.0 ± 5	90.9 ± 14*,#	68.8 ± 13*

Phase III										
Dietary regimen	*N*	Week 1			Week 2			Week 3		
		Caloric Intake (kcal/day)	Fluid Intake (ml/day)	Urine Output (ml/day)	Caloric Intake (kcal/day)	Fluid Intake (ml/day)	Urine Output (ml/day)	Caloric Intake (kcal/day)	Fluid Intake (ml/day)	Urine Output (ml/day)
GCS‐GCS	9	62.6 ± 6	32.0 ± 5	18.7 ± 4	63.5 ± 6	31.8 ± 5	18.3 ± 4	64.3 ± 5	32.2 ± 4	19.0 ± 4
FCS‐GHS	9	45.9 ± 5*	65.5 ± 14*,†	49.9 ± 15*,†	54.4 ± 4*	72.7 ± 15*	56.3 ± 15*	57.1 ± 4†	79.7 ± 18*	65.9 ± 18*
FHS‐GHS	8	50.1 ± 7*	58.2 ± 10*,†	41.8 ± 7*,†	56.1 ± 6*	69.6 ± 11*	52.7 ± 10*	59.9 ± 6	76.6 ± 12*	60.6 ± 11*
FHS‐FHS	9	60.5 ± 5	68.3 ± 19*	63.9 ± 6	86.2 ± 16*	68.6 ± 18*	66.4 ± 8	91.6 ± 18*	71.4 ± 13*

*Note*: Values are mean ± SE. Average caloric intakes by week have been reported previously (Levanovich et al., 2021) and were calculated using caloric profiles of 3.98 kcal/g and 3.61 kcal/g for 0.4% and 4% salt chows, respectively.

* *p* < 0.05 vs GCS‐GCS.

† *p* < 0.05 vs. FHS‐FHS.

# *p* < 0.01 vs week 1.

Fluid intake increased significantly in week 2 and remained higher in week 3 compared with week 1 in the FHS group. Urine output also increased over time in this group but did not achieve significance (Table 2). Notably, fluid intake and urine volumes in the FHS group were two to three times the amounts observed in the GCS and FCS groups throughout Phase I (*p* < 0.05). Likewise, fluid intake and urine volumes were significantly greater in each of the three groups fed 4% salt diets during Phase III compared with the GCS‐GCS group (*p* < 0.05). During week 1 of Phase III, fluid intake and urine volumes were also higher in the FHS‐FHS rats compared with FCS‐GHS and FHS‐GHS rats (*p* < 0.05).

### 3.2 Sodium and potassium balances

**FIGURE 3:**
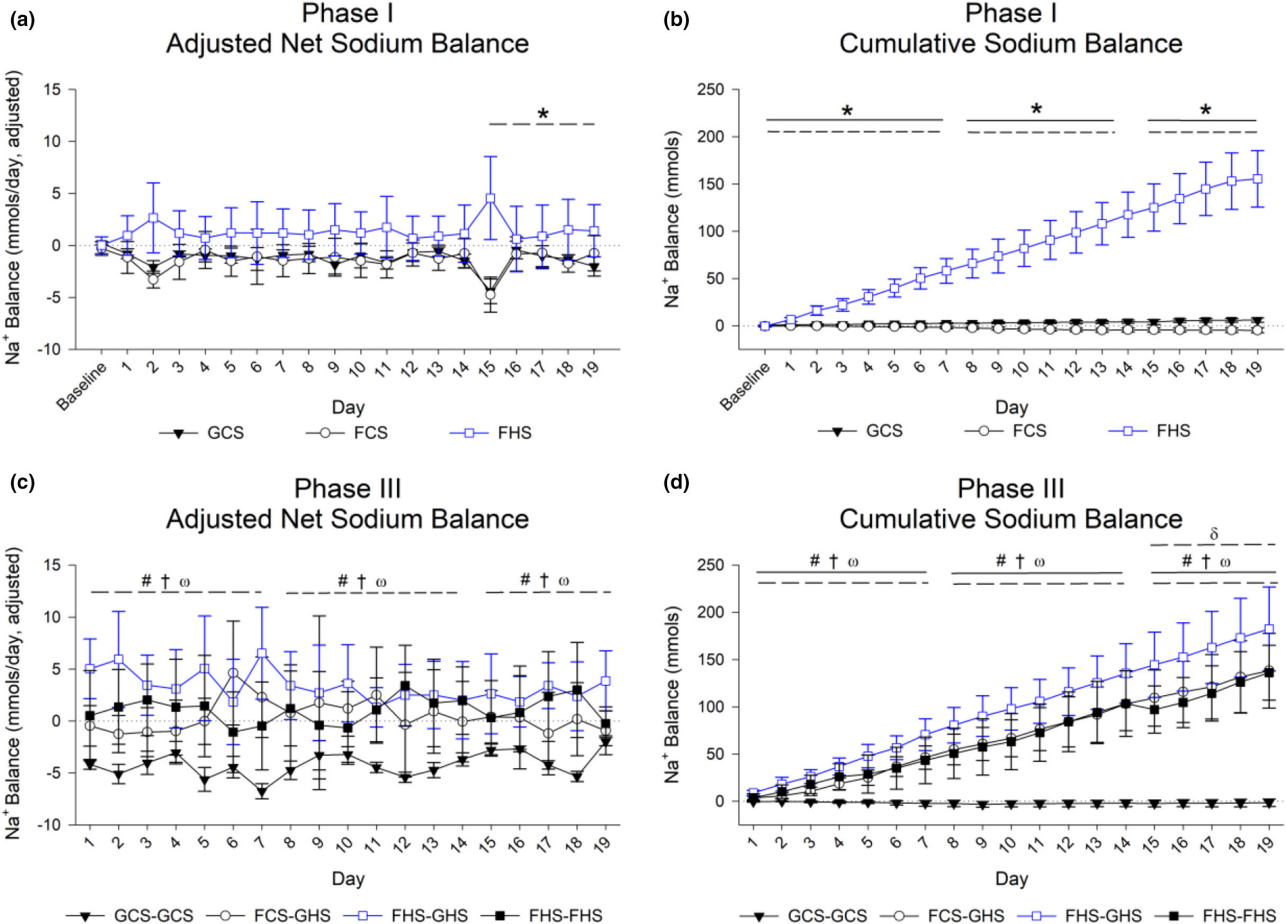
Bodily sodium balance. Measurements obtained via flame photometry analysis of urine samples collected daily. Net measurements were statistically adjusted for daily calorie intake and urine volume. Profile analysis results are annotated: a broken line indicates a significant main effect of group (*p* < 0.02; average between‐group differences in the week), and a solid line indicates significant group × day interaction (*p* < 0.02; between‐group differences in the change). (a) Phase I net sodium balances—FHS had average elevation relative to GCS in week 3. (b) Phase I cumulative sodium balance—FHS had a consistently higher average and a faster rate of change compared to GCS each week. (c) Phase III net sodium balances—all experimental groups had stable, higher values than GCS‐GCS each week. (d) Phase III cumulative sodium balance—all experimental groups had higher average cumulative sodium and a faster rate of change compared with GCS‐GCS each week. In week 3, FHS‐GHS had higher average values compared with FHS‐FHS. Values are indicated as mean ± 2 SE; n as indicated per group in Table 2. Symbols denote post hoc group comparisons: Phase 1, *p < 0.05 FHS vs. GCS controls; Phase III, #*p* < 0.05 FCS‐GHS vs. GCS‐GCS, †*p* < 0.05 FHS‐GHS vs. GCS‐GCS, ω*p* < 0.05 FHS‐FHS vs. GCS‐GCS, δ*p* < 0.05 FHS‐GHS vs. FHS‐FHS.

The lower caloric intake during the initial days of transition from 0.4% to 4% diets impacted the overall sodium intake and outputs. Likewise, the urine outputs also varied by ~3‐fold compared with the GCS‐GCS group. Thus, the data for net sodium intake were statistically adjusted for caloric and urine volume (Figure 3). The unadjusted data are provided in the Appendix (Figure S1).

During Phase I, and consistent with the lower overall consumption of chow in the initial days of week 1, sodium intake was only ~8‐ to 9‐fold greater in the FHS group compared with the GCS and FCS groups. By week 2, sodium intake by the FHS rats achieved a 10‐fold greater sodium intake than that consumed by GCS and FCS (Table 3). This pattern was recapitulated when FCS‐GHS and FHS‐GHS were transitioned from 0.4% sodium chow during Phase II to 4% sodium chow in Phase III. Except for week 1 of Phase III due to lower chow intake in the FCS‐GHS and FHS‐GHS rats, potassium intakes were similar across all groups.

**TABLE 3:** Sodium and potassium intakes and excretion rates during Phase I and Phase III

Phase I													
Dietary regimen	*N*	Week 1				Week 2				Week 3			
		Consumption (mmols/day		Excretion Ux × V (mmols/day)		Consumption (mmols/day		Excretion Ux × V (mmols/day)		Consumption (mmols/day		Excretion Ux × V (mmols/day)	
		Na^+^	K^+^	Na^+^	K^+^	Na^+^	K^+^	Na^+^	K^+^	Na^+^	K^+^	Na^+^	K^+^
GCS	9	3.0 ± 0.3	4.9 ± 0.5	2.4 ± 0.1	3.9 ± 0.1	3.0 ± 0.1	4.8 ± 0.1	2.7 ± 0.2	4.1 ± 0.3	2.9 ± 0.1	4.7 ± 0.1	2.6 ± 0.2	4.1 ± 0.2
FCS	9	2.7 ± 0.1	4.3 ± 0.2	3.0 ± 0.2	4.1 ± 0.2	3.0 ± 0.1	4.8 ± 0.1	3.4 ± 0.1	4.6 ± 0.2	3.2 ± 0.3	5.2 ± 0.6	2.8 ± 0.2	4.3 ± 0.1
FHS	17	4.0 ± 0.2	3.6 ± 0.2	5.0 ± 0.1	4.7 ± 0.2	5.0 ± 0.1	3.6 ± 0.6

Phase III													
Dietary regimen	*N*	Week 1				Week 2				Week 3			
		Consumption (mmols/day		Excretion Ux × V (mmols/day)		Consumption (mmols/day		Excretion Ux × V (mmols/day)		Consumption (mmols/day		Excretion Ux × V (mmols/day)	
		Na^+^	K^+^	Na^+^	K^+^	Na^+^	K^+^	Na^+^	K^+^	Na^+^	K^+^	Na^+^	K^+^
GCS‐GCS	9	2.8 ± 0.1	4.5 ± 0.1	3.1 ± 0.4	4.3 ± 0.7	2.9 ± 0.1	4.6 ± 0.1	2.7 ± 0.1	4.3 ± 0.2	2.8 ± 0.1	4.6 ± 0.1	2.6 ± 0.1	4.2 ± 0.2
FCS‐GHS	9	22.1 ± 0.8*,†	3.6 ± 0.1*,†	15.8 ± 1.3*,†	3.3 ± 0.6	26.2 ± 0.7*	4.2 ± 0.1†	4.1 ± 0.9	27.5 ± 0.6*	4.5 ± 0.1	20.4 ± 1.4*	4.9 ± 1.3
FHS‐GHS	8	23.3 ± 1.2*	3.8 ± 0.2*,†	13.2 ± 0.6*,†	3.7 ± 0.6	26.0 ± 0.8*	4.2 ± 0.1†	16.7 ± 1.0*,†	4.0 ± 0.2	27.7 ± 0.6*	4.5 ± 0.1	18.2 ± 1.3*	4.6 ± 0.6
FHS‐FHS	9	27.5 ± 0.8*	4.4 ± 0.1	20.5 ± 1.6*	4.4 ± 0.3	29.0 ± 0.3*	4.7 ± 0.1	20.4 ± 1.6*	4.3 ± 0.3	29.9 ± 1.0*	4.9 ± 0.2	20.7 ± 1.7*	4.5 ± 0.2

*Note*: Values are mean ± SE. Average sodium intake by week was reported previously (Levanovich et al., 2021).

* *p* < 0.05 vs GCS (Phase I) or GCS‐GCS (Phase III).

† *p* < 0.05 vs. FHS‐FHS.

Urinary sodium excretion rates were greater in FHS rats compared with GCS and FCS during Phase I. Likewise, the high salt diets in Phase III resulted in higher urinary sodium excretion in all three groups compared with the GCS‐GCS group (Table 3). During weeks 1 to 3 of Phase I, sodium excretion by the GCS and FCS groups was comparable to intake, whereas sodium excretion by the FHS rats was significantly lower than the amount of sodium consumed each day. In Phase III, this pattern was again observed wherein the GCS‐GCS rats' daily sodium intake and output were similar. Nonetheless, sodium excretion rates by the FCS‐GHS, FHS‐GHS, and FHS‐FHS groups were consistently and significantly lower than their sodium consumption (Table 3).

The disparities in sodium handling are reflected in the net sodium balances. Notably, when the net sodium balance is adjusted for caloric intake (since sodium was provided in the chow) and urine flow rate, groups differed in the day‐to‐day net sodium balance only during week 3 of Phase 1 (*p* < 0.02) due to elevations in the FHS group compared with the GCS group (Figure 3a). When no adjustments were made, the net sodium balance was higher throughout Phase 1 after day 2 (Figure S1‐A1 and A2). Cumulative sodium balance was greater in FHS rats compared with GCS or FCS rats beginning with day 2 of Phase 1, and the faster rate of increase relative to GCS across all weeks (Figure 3b). The latter was evident in the unadjusted data as well (Figure S1‐B1 and B2).

During Phase III, GCS‐GCS rats displayed an unadjusted zero net sodium balance, whereas the other groups each displayed positive net sodium balances (Figure S1‐C1 and C2). The nonadjusted net sodium balances were significantly higher in FCS‐GHS, FHS‐GHS, and FHS‐FHS groups vs the GCS‐GCS group from day 1 and remained significantly higher throughout this phase (Figures S1‐C3 and S1‐C). Caloric and urine flow adjustments did not alter this appraisal (*p* < 0.02; Figure 3C). Groups differed in the rate of change in cumulative sodium balance over the 3 weeks (*p* < 0.02) which was driven by the stable levels in the GCS‐GCS group. A pattern of between‐group differences emerged over the 3 weeks. Figure 3D shows that all groups fed 4% sodium chow were equivalent during weeks 1 and 2 and greater than GCS‐GCS rats (*p* < 0.01). By week 3, the FHS‐GHS group displayed a higher cumulative sodium balance than the FHS‐FHS group on average (*p* < 0.03). The unadjusted data also demonstrate that all groups fed a 4% salt chow diet displayed a cumulative increase relative to the stable low levels of the GCS‐GCS group across all 3 weeks resulting in significantly higher average cumulative sodium levels relative to GCS‐GCS each week. By week 3, the FHS‐GHS rats additionally had significantly higher average sodium compared with FHS‐FHS rats (*p* < 0.05; Figure S1‐D1 and D2).

### 3.3 Blood pressure

**FIGURE 4:**
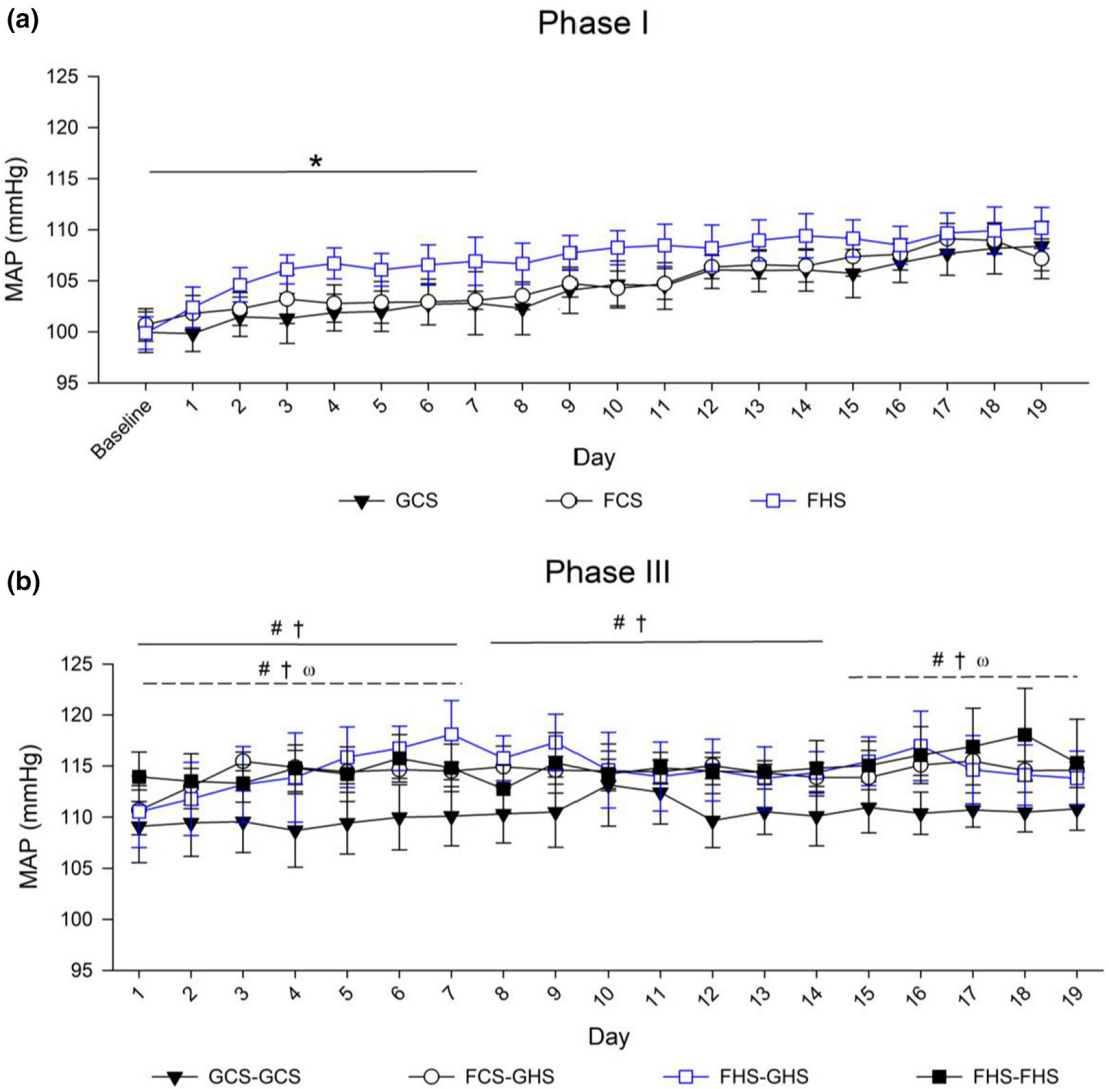
Mean arterial pressure (MAP) across phases. MAP was assessed by telemetry during Phase I (a) and Phase III (b). Values are indicated as mean ± 2 SE, N as indicated per group. Profile analysis results are annotated: a broken line indicates a significant main effect of the group (*p* < 0.02; average between‐group differences in the week), and a solid line indicates significant group × day interaction (*p* < 0.02; between‐group differences in the change). (a) Phase I—FHS demonstrated a faster rate of increase in mean arterial pressure in week 1. (b) Phase III—All experimental groups had higher average measurements compared with GCS‐GCS in week 1 and week 3; FHS‐FHS displayed stable elevations over all weeks, whereas FCS‐GHS and FHS‐GHS had a faster rate of change compared with GCS‐GCS in weeks 1 and 2. Symbols denote post hoc group comparisons: Phase I, *p < 0.05 FHS vs. GCS controls; Phase III, #*p* < 0.05 FCS‐GHS vs. GCS‐GCS, †*p* < 0.05 FHS‐GHS vs. GCS‐GCS, ω*p* < 0.05 FHS‐FHS vs. GCS‐GCS.

As the rats matured during Phase I, MAP rose across groups (*p* < 0.02). The addition of high salt to a fructose diet led to a faster rate of increase in MAP in FHS rats that was significantly elevated after 4 days compared with GCS and FCS rats (Figure 4a). MAP remained elevated in FHS rats for 7 days at which time MAP became statistically comparable to that of the other groups. Whereas final MAP did not differ among the groups at the end of Phase I, the overall change between final and baseline values in the FHS rats (10.5 ± 2.8 mmHg) was statistically greater than either GCS 6.7 ± 2.1 mmHg (*p* < 0.01) or FCS rats 7.1 ± 2.9 mmHg (*p* < 0.05).

In week 1 of Phase III, groups differed in the pattern of change (*p* < 0.02): FCS‐GHS and FHS‐GHS groups demonstrated an increase in MAP compared with stable high MAP in the FHS‐FHS group and stable low MAP in the GCS‐GCS group (*p* < 0.05; Figure 4b). By week 3 of Phase III, MAPs were consistently higher in the three groups given 4% sodium in the diet although statistical significance varied on a day‐to‐day analysis. Compared with blood pressures at the beginning of the study, however, MAP overall rose higher in the FCS‐GHS (14.6 ± 0.9 mmHg), FHS‐GHS (14.6 ± 1.4 mmHg), and FHS‐FHS groups (17.8 ± 2.0 mmHg) than the GCS‐GCS group (9.7 ± 1.0 mmHg; *p* < 0.05). Systolic blood pressures displayed a similar overall pattern, but diastolic pressures and heart rates were similar among the groups and have been reported elsewhere (Levanovich et al., 2021).

### 3.4 Renal function

**FIGURE 5:**
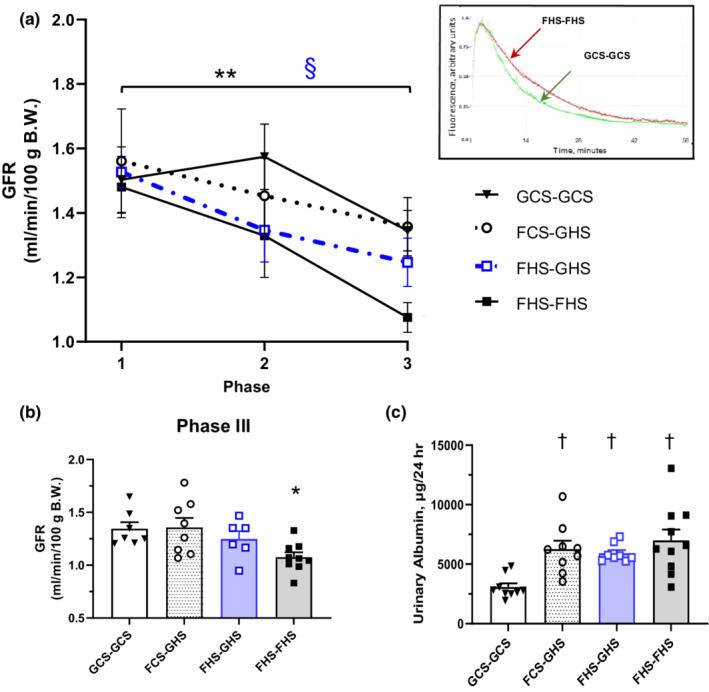
Glomerular filtration rate (GFR) and albumin excretion. (a) GFR at the end of Phases I, II, and III in each of the groups: GCS‐GCS (*n* = 7), FCS‐GHS (*n* = 8), FHS‐GHS (*n* = 6), and FHS‐FHS (*n* = 8); *p < 0.05 vs GCS‐GCS. Inset depicts typical curves from one GCS‐GCS rat and one FHS‐FHS rat at the end of Phase III; §*p =* 0.03 across Phases I to III for FHS‐GHS; ** *p* < 0.005 across Phases I to III for FHS‐FHS. (b) GFR at the end of Phase III for each of the four groups; *p* < 0.05 vs GCS‐GCS. (c) Urinary albumin excretion over 24 h in each of the groups at the end of Phase III: GCS‐GCS, *n* = 9; FCS‐GHS, *n* = 9; FHS‐GHS, *n* = 8; FHS‐FHS, *n* = 9; † *p* < 0.02 vs GCS‐GCS.

GFR did not differ among groups at the end of either Phase I or Phase II. Notably, the FHS‐FHS rats exhibited an ~30% decline in GFR from Phase I to Phase III (*p* < 0.005). Likewise, the FHS‐GHS group also exhibited a significant decline over the three phases (*p* = 0.03). Both the GCS‐GCS and FCS‐GHS groups displayed no significant change over the course of the three phases. At the end of Phase III, GFR was significantly lower in the FHS‐FHS rats compared with that of GCS‐GCS rats (Figures 5a,b). Although FCS‐GHS groups did not display a significant decline in GFR over time, 24 h urinary albumin excretion was significantly elevated in all three groups fed fructose during Phase I compared with the GCS‐GCS group (Figure 5c).

### 3.5 Metabolic and hormonal parameters

Metabolic and hormonal values for rats in each of these groups have been reported elsewhere (Levanovich et al., 2021). In summary, at the completion of Phase III and just prior to harvesting tissues, fasting glucose and insulin levels did not differ among the groups; however, the glucose:insulin ratio as in the index of insulin sensitivity was significantly lower in the FHS‐GHS and FHS‐FHS groups compared with either GCS‐GCS or FCS‐GHS groups (*p* < 0.05). Despite the elevated blood pressures, PRA was appropriately suppressed in the FCS‐GHS rats (0.66 ± 0.12 ngAngI/ml/h) vs GCS‐GCS rats (1.82 ± 0.20 ngAngI/ml/h; *p* < 0.05), but not the FHS‐GHS (1.35 ± 0.28 ngAngI/ml/h) or FHS‐FHS rats (1.09 ± 0.29 ngAngI/ml/h).

### 3.6 Histology

**FIGURE 6:**
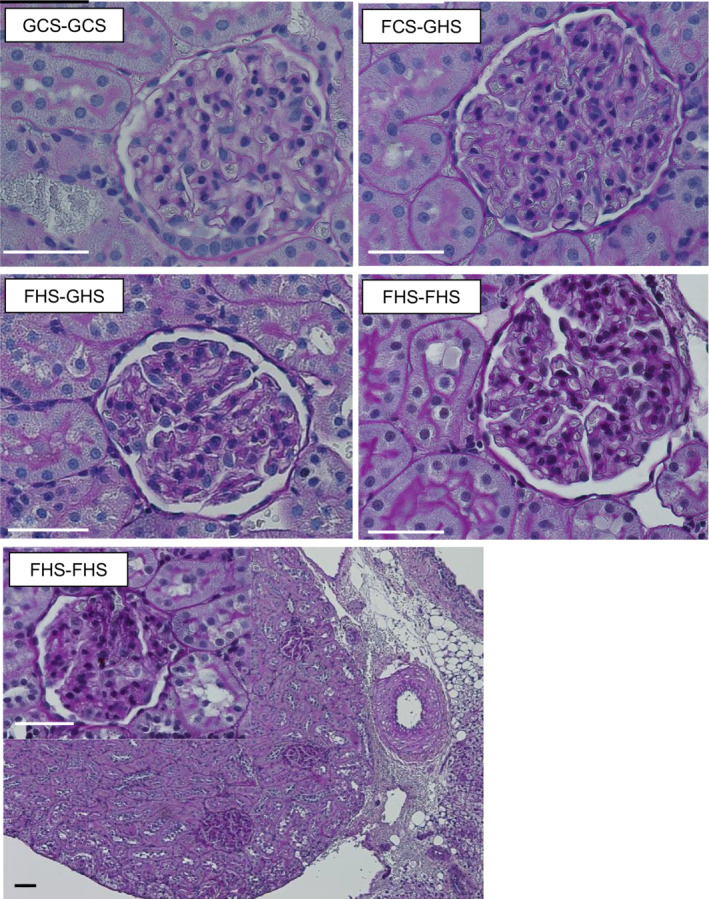
Representative renal histology. Periodic acid Schiff stained tissue from each group of rats (original 60× magnification). GCS‐GCS rats displayed normal glomerular morphology. FCS‐GHS and FHS‐GHS rats had several glomeruli with mesangial hypercellularity. Tissue from FHS‐FHS rats displayed the most prominent mesangial expansion and hypercellularity. The bottom panel shows 20× magnification of renal tissue and blood vessel with an inset of glomerulus at 60× magnification from an FHS‐FHS rat. Quantitative histomorphometry was not performed. The scale bars represent 50 μm for each image.

The study was not designed for quantitative histopathologic assessment. Figure 6 shows typical glomerular histology from kidneys in each of the four groups. Glomeruli from the GCS‐GCS group were normal. Fructose‐fed rats tended to display mesangial expansion and hypercellularity which was most prominent in the FHS‐FHS group. Tubular morphology was not remarkable in any of the groups.

## 4 DISCUSSION

The major finding of the present study is that high dietary fructose either alone or together with high sodium consumption during the critical preadolescent and adolescent periods resulted in salt‐sensitive blood pressure elevation later in life. Despite resuming a diet devoid of fructose and with normal salt intake during young adulthood, high fructose intake during this early critical period is predisposed to sodium retention in maturity. Urinary albumin excretion in maturity was elevated in all groups that ingested fructose during this critical period. Moreover, when high fructose and high salt were ingested throughout adolescence into young adulthood and maturity, the renal function also declined. Together, these data show that dietary intake of fructose during this critical developmental period can exert lasting effects rendering individuals susceptible to hypertension later in life.

Induction of hypertension in adult outbred Sprague Dawley rats typically requires extended periods of exposure to diets with a very high salt content, typically 8% sodium chloride (Gu et al., 2008; Osborn & Hornfeldt, 1998), or selective breeding, hormonal, surgical, or genetic modification (Dietz et al., 1982; Elijovich et al., 2016; Folkow & Svanborg, 1993; Ise et al., 1998; Jelinek, 1972; Winternitz & Oparil, 1982; Wyss et al., 1994). Furthermore, Sprague Dawley rats consuming a fructose diet as high as 66% alone are insulin resistant but do not develop high blood pressure when measured by telemetry, although they do display elevated systolic blood pressures by tail‐cuff plethysmography (D'Angelo et al., 2005). In contrast, when moderately high fructose proportional to that observed in the upper quintile of human populations is combined with high salt intake in adult rats, hypertension results (Cabral et al., 2014; Gordish et al., 2017; Levanovich et al., 2021; Soncrant et al., 2018; Zenner et al., 2018). Taken together, these observations highlight the importance not only of the underlying species, strain, and model when studying the impact of diet on blood pressure but also of the importance of other food components and modalities for assessing blood pressure.

The present study used chronic telemetry‐based hemodynamic recordings to evaluate the impact of fructose with normal as well as high sodium on blood pressure in outbred Sprague Dawley rats. Blood pressure typically increases in both normal adolescent humans (Ataei et al., 2016) and rats at 5–7 weeks of age (Hom et al., 2007). A gradual increase in blood pressure was evident in all groups during Phase I; however, high sodium intake ingested along with fructose during this period led to a more rapid rise in MAP, whereas high fructose intake alone did not change the trajectory of blood pressure. Notably, all three groups achieved the same MAP at the end of this phase. Clearly, the fructose‐high salt group exhibited a positive cumulative and a small but measurable net sodium balance, even when corrected for caloric intake and urine volume. The group fed only high fructose remained in zero cumulative and net sodium balance similar to that of the control glucose‐fed rats. Although fructose increases sodium absorption by the gut (Barone et al., 2009; Singh et al., 2008), fecal excretion of sodium is unchanged and any excess in sodium intake would have required excretion via the urine (Gordish et al., 2017). The positive net sodium balance in the fructose‐fed high salt group would thus be expected to have an expanded extracellular fluid volume compared with the other two groups.

Increases in extracellular volume cannot augment arterial pressure indefinitely. Counter‐regulatory measures such as baroreflex suppression of sympathetic efferent activity, hypertension‐induced inhibition of the renin‐angiotensin system function, and enhanced nitric oxide activity among other factors should come into play to mitigate increases in blood pressure. Dietary fructose has been shown to impair each of these responses to high salt intake (Gordish et al., 2017; Klein & Kiat, 2015; Palanisamy & Venkataraman, 2013; Soncrant et al., 2018; Zenner et al., 2018). Pressure natriuresis would also be expected to occur. However, dietary fructose enhances the sensitivity of the proximal tubule to angiotensin II (Ang II) such that renal tubular sodium reabsorption is increased, thereby attenuating pressure natriuresis (Cabral et al., 2014; Gonzalez‐Vicente et al., 2018; Gonzalez‐Vicente, Cabral, et al., 2019; Yang et al., 2020). Overall, it is reasonable to infer that young rats fed fructose and high salt, such as those at the end of Phase I, still possess the ability to counter‐regulate the impact of a positive sodium balance so as to maintain blood pressure similar to that of glucose‐fed rats on control salt diet. Such a mechanism has been invoked in the age‐dependent salt sensitivity of Dahl salt‐sensitive (SS) rats (Dobesova et al., 1995). Additional studies will be needed to determine which, if any, of these or other mechanisms are involved in maintaining blood pressure homeostasis at this stage.

High fructose consumption, regardless of whether it was combined with a control or a high salt diet in early life, resulted in higher blood pressures when the rats were challenged with a high salt diet in maturity. This observation is consistent with our initial hypothesis that rats fed fructose when they are at 5–7 weeks of age would exhibit salt‐sensitive blood pressure later in life. Importantly, when adult outbred Sprague Dawley rats were given 20% glucose with a 4% sodium chloride diet for 3 weeks without prior exposure to fructose, blood pressure was not elevated (Komnenov et al., 2020). Thus, a high fructose diet during the critical young phase can predispose to salt‐sensitive blood pressure elevation. Although the elevation in mean arterial pressure may seem small (~5 mmHg) compared with the GCS‐GCS controls, it is nonetheless significant. Moreover, since diastolic blood pressure is similar in all four groups, it is the substantial difference in systolic blood pressure (~8–9 mmHg) that is driving hypertension (detailed values provided in our previous publication (Levanovich et al., 2021)). Systolic hypertension is consistent with diminished vascular compliance exhibited by fructose‐fed animals (Levanovich et al., 2021). Importantly, epidemiological studies estimate that a reduction in systolic blood pressure of 5 mmHg in humans reduces the risk of mortality from stroke by 14% and coronary heart disease by 9% (Appel, 2017).

Cumulative sodium balance during Phase III was greater in all three groups fed fructose in Phase I. The greater increase in the FHS‐GHS rats at first seemed somewhat paradoxical. Two potential explanations may account for this observation. First, the FHS‐GHS group started Phase III with a slightly greater cumulative sodium balance on day 1 (13 mmol) compared with either FCS‐FHS (2.8 mmol) or FHS‐FHS (4.0 mmol) as well as the control group (−0.2 mmol; Figure S1 D2). As a consequence, although the slopes of the cumulative sodium balance were similar in the groups fed 4% sodium, the FHS‐GHS had a higher overall cumulative sodium balance at the end of Phase III. Second, the rats fed fructose plus a high salt diet throughout the study may have more well‐developed compensatory mechanisms over time. Notably, net sodium balance was lower in the FHS‐FHS group during week 1 of Phase III. We speculate that the FHS‐FHS group had sufficient time and sodium retention over Phases I and II such that those rats were better able to escape from the effects of aldosterone. In contrast, the FHS‐GHS group had been on GCS during Phase II, such that the RAS system may not have been suppressed as well during week 1 of Phase III and/or that escape from aldosterone was not fully engaged until later in Phase III. Consistent with this possibility was the observation that PRA in the FHS‐GHS rats was 27% higher albeit not significantly different, than in the FHS‐FHS rats, by the end of Phase III (Levanovich et al., 2021). Nevertheless, the findings compared with the GCS‐GCS control group indicate that a diet high in fructose with or without a concurrent high salt diet during a limited period in early life results in limited excretion of sodium when presented with high salt intake in maturity.

Previous in vivo and ex vivo studies in adult rats have shown that either short term or chronic fructose feeding, as well as acute perfusion with fructose, increases the expression and activity of fructose, sodium, and chloride transport systems across the intestinal and renal epithelia. The osmotic gradient that develops in the intestine is important for absorption and initial increases of extracellular volume while sodium reabsorption mechanisms within the kidney serve to maintain extracellular expansion (Antoine et al., 1997; Ares et al., 2019; Barone et al., 2009; Cabral et al., 2014; Queiroz‐Leite et al., 2012; Singh et al., 2008). In addition, rats fed fructose and high salt did not appropriately suppress PRA either in the present or previous studies (Gordish et al., 2017; Levanovich et al., 2021; Soncrant et al., 2018). Ang II enhances proximal tubular sodium reabsorption, an effect that is augmented in tubules from fructose‐fed rats (Cabral et al., 2014; Gonzalez‐Vicente et al., 2018; Gonzalez‐Vicente, Cabral, et al., 2019; Yang et al., 2020) and is an independent determinant of hypertension in response to salt intake (Chiolero et al., 2000). Efferent renal sympathetic nerve activity which is also heightened with fructose feeding (Komnenov et al., 2019; Soncrant et al., 2018) can increase sodium reabsorption by several nephron segments (DiBona, 1985). In contrast to the peripheral RAS system, high salt intake activates intrarenal RAS. Fructose independently augments the expression of components of intrarenal RAS, and intrarenal RAS has been implicated in salt sensitivity (Majid et al., 2015; Xu et al., 2017; Yokota et al., 2018).

The induction of inflammatory cytokines and reactive oxygen species within the kidney have also been implicated in sodium retention and hypertension in this model (Komnenov et al., 2020; Zenner et al., 2018). Oxidative stress can further induce vascular dysfunction, tubule sodium reabsorption, autonomic dysregulation, and renin secretion (Gonzalez‐Vicente & Garvin, 2017; Gonzalez‐Vicente, Hong, et al., 2019; Grillo et al., 2019; Thorup & Persson, 1994). The accumulation of sodium within the skin may mitigate volume expansion but result in stimulating immune‐mediated sympathoexcitation which, in turn, increases vascular resistance (Balafa & Kalaitzidis, 2021; Schatz et al., 2017). Impaired generation of renal nitric oxide is involved in other models of salt‐sensitive hypertension such as the Dahl SS rat (Hong et al., 2021; Zou & Cowley Jr., 1999) as well as adult rats fed fructose plus high salt (Gordish et al., 2017). Uric acid is a known by‐product of fructose catabolism and has been shown to decrease nitric oxide availability and impair mitochondrial respiration. The reduced mitochondrial respiration ultimately results in preferential glycolysis and the formation of reactive oxygen species (De Becker et al., 2019), both of which have been implicated in dysregulated extracellular matrix formation and cell proliferation/apoptosis via the Warburg effect (Gherghina et al., 2022; Lunt & Vander Heiden, 2011). Whether prior exposure to high fructose during youth alters these or other mechanisms later in life will need further study.

Alterations in baroreceptor function have been identified in several models of hypertension and can modulate both blood pressure and renal sodium excretion (Mark, 1991; Mark et al., 1987; Maybaum et al., 1998; Rossi et al., 2019; Shimoura et al., 2017). Impaired baroreflex function may serve to increase total peripheral resistance, cardiac function, or directly influence the RAS. Indeed, renal sympathetic activity is increased in adult rats fed fructose and high salt diet and directly induces renin secretion (Farah et al., 2007; Soncrant et al., 2018). Interestingly, inhibition of RAS in very young male Sprague Dawley rats leads to increased RAS activation later in life (Bualeong et al., 2019). This is consistent with the current observation that preadolescent rats given fructose with 0.4% sodium were able to suppress PRA when challenged with 4% sodium in maturity. In contrast, fructose administered with 4% salt which should have suppressed PRA in the young rats resulted in failure to inhibit renin secretion with a high salt diet later in life. Since maternal fructose has been shown to induce epigenetic changes in several proteins in the RAS cascade as well as renal sodium transporters (Cho & Kim, 2020; Seong et al., 2019), it is conceivable that ingestion of fructose with or without high salt in early life may also result in epigenetic alterations and changes in protein activities. As with studies in prenatal programming of hypertension, the present findings provide a strong rationale for a rigorous investigation into the mechanisms involved in programming that may occur with dietary intake in youth.

### 4.1 Limitations

Blood was obtained in awake animals so as not to have the confounding influence of anesthesia as well as providing careful attention to the volume and rate of sampling to avoid inducing hypotension, either of which is known to influence PRA and the RAS cascade. Thus, sufficient plasma was not available to assess Ang II, aldosterone, or uric acid in addition to the electrolytes, glucose, PRA, and insulin levels. In addition, the study would benefit from rigorous histologic scoring of the renal tissue. In the interest of providing analysis that is as accurate and reproducible as possible, we elected to show representative images for a qualitative impression and would welcome collaboration with individuals skilled in renal histopathology.

### 4.2 Summary

Fructose ingestion by male Sprague Dawley rats at a level comparable to that in the upper quintile of human consumption alone or in conjunction with a high salt diet during a limited, critical phase early in life leads to positive sodium balance, salt‐sensitive hypertension, and albuminuria when challenged with salt in maturity. Combined intake of fructose and a high salt diet throughout adolescence, young adulthood, and into maturity also results in a substantial decline in renal function.

#### 4.2.1 Perspectives

The current study certainly has obvious implications for the potential impact of high fructose with or without high salt intake in adolescents on blood pressure and renal function later in life. In contrast to preclinical models such as the rodent, control of other dietary components that may impact blood pressure alone or in conjunction with sodium is more challenging in human studies. Dietary studies in human populations largely focus on one component. The present data strongly suggest that caution be exerted in cross‐sectional studies of fructose intake (and perhaps other dietary components) since ongoing fructose intake was not required to induce salt‐sensitive blood pressure. Historical intake, especially in youth, may exert an effect that confounds subsequent analyses after individuals may have altered their diets. Notably, adolescents reportedly have the highest intake of fructose (Marriott et al., 2009). With these caveats in mind, it is noteworthy that a longitudinal study in humans revealed that fructose consumption greater than 7.4% of total caloric intake was associated with higher blood pressure, cardiovascular disease, and increased serum creatinine (Bahadoran et al., 2017). Data exist showing that the negative impact may be even greater in individuals of African American descent (DeChristopher et al., 2020).

## AUTHORS’ CONTRIBUTION

Peter E. Levanovich: performed and analyzed experiments, wrote manuscript; Ana Daugherty: statistical analyses, edited manuscript; Dragana Komnenov: edited manuscript; Noreen F. Rossi: conceived experimental design, performed, and analyzed experiments, edited manuscript.

## Supporting information


Data S1
