# Corrigendum: Dietary fructose and high salt in young male Sprague‐Dawley rats induce salt‐sensitive changes in renal function in later life

**DOI:** 10.14814/phy2.15647

**Published:** 2023-03-22

The author of the article by Noreen F. Rossi (2022) noted an error in Figure 1 of their paper. The boxes showing fructose high salt and glucose high salt in the protocol at the very bottom of Phase III were reversed. The text in the methods and otherwise are correct regarding the groups.

The correct figure is as follows:

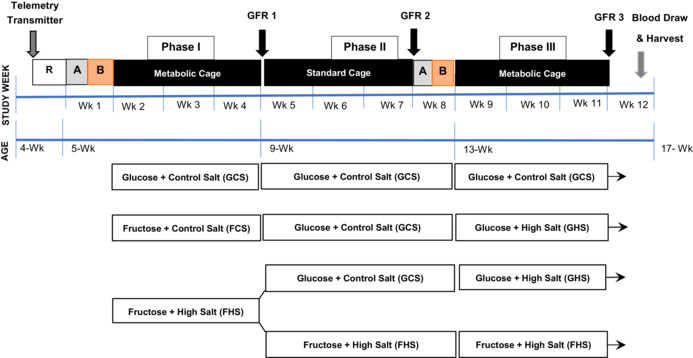

## References

[phy215647-bib-0001] Levanovich, P. E. , Daugherty, A. M. , Komnenov, D. , & Rossi, N. F. (2022). Dietary fructose and high salt in young male Sprague Dawley rats induces salt‐sensitive changes in renal function in later life. Physiological Reports, 10, e15456. 10.14814/phy2.15456 36117446 PMC9483717

