# Supplementary material for: Dietary fructose and high salt in young male Sprague Dawley rats induces salt‐sensitive changes in renal function in later life
Source: Physiol Rep. 2022 Sep 19;10(18):e15456. doi: 10.14814/phy2.15456 (PMC9483717; doi:10.14814/phy2.15456)
Supplement: Supplementary file 1 — Data S1 [file PHY2-10-e15456-s001.docx]

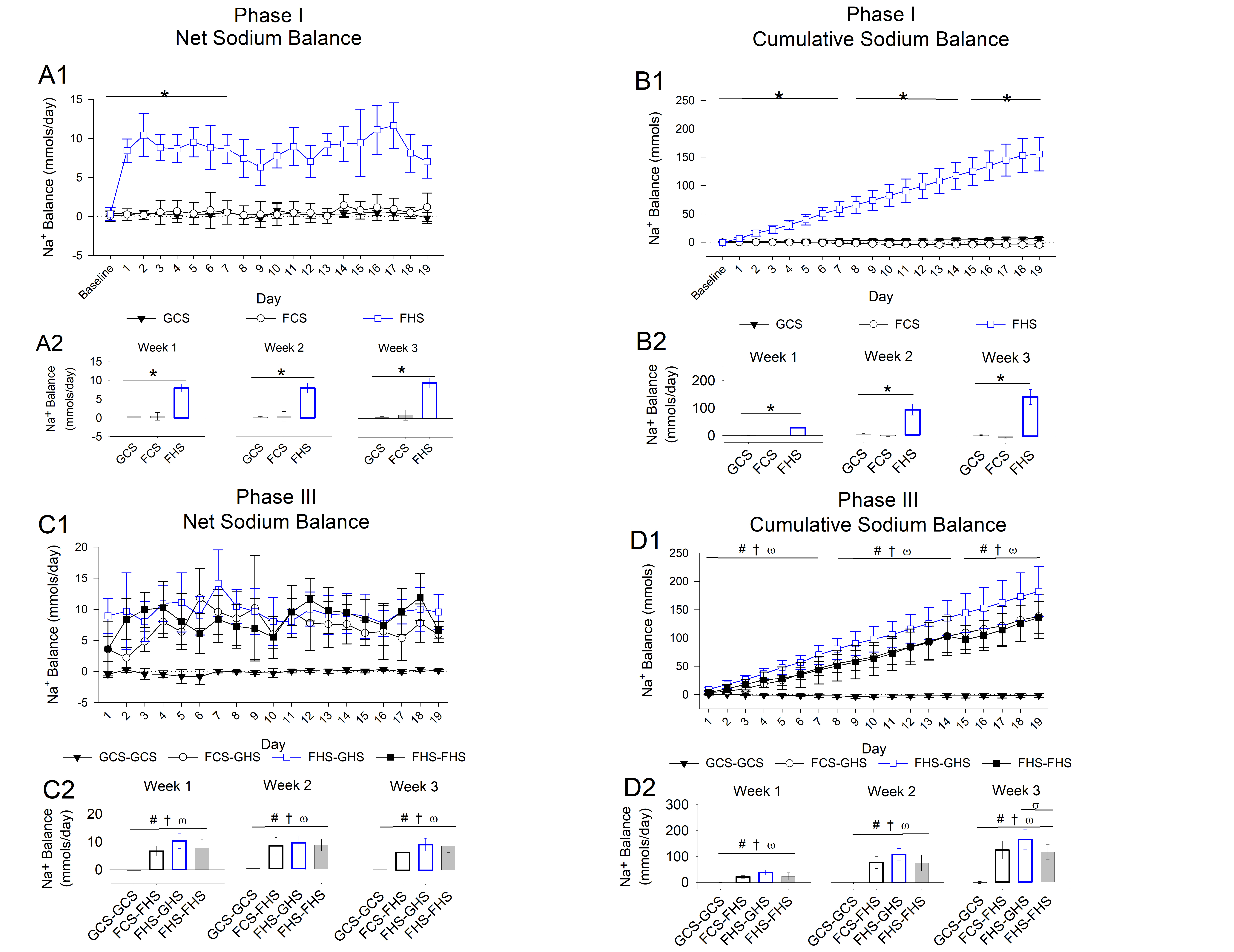


(mmols)

(mmols)

**Figure S1: Unadjusted Bodily Sodium Balance.** Measurements obtained via flame photometry analysis of urine samples collected daily. Profile analysis results are annotated (panels A1, B1, C1, D1) as significant Group × Day interaction indicating significant group differences in the pattern of change per week (*p* < 0.02). The analysis accounts for the main effect of Group (panels A2, B2, C2, D2), in which groups differ in average measurements per week. Phase I net sodium balances: (A1) FHS had faster rate of change relative to GCS in week 1, and (A2) FHS had on average higher values each week as compared to GCS. Phase I Cumulative Sodium Balance: (B1) FHS had a faster rate of increase relative to GCS across all weeks, which resulted in (B2) higher average levels compared to GCS in each week. Phase III Net Sodium Balances—(C1) all groups demonstrated statistically equivalent rates of change over each week, but (C2) all groups fed a 4% salt chow had significantly higher average net sodium balance as compared to GCS-GCS in each week. Phase III Cumulative Sodium Balance—(D1) All groups fed a 4% salt chow diet demonstrated a cumulative increase relative to the stable low levels of GCS-GCS across all weeks, which resulted in (D2) significantly higher average levels relative to GCS-GCS each week. By week 3 FHS-GHS additionally had significantly higher average sodium as compared to FHS-FHS. Values are indicated as mean ± 2 SE; n as indicated per group in Table 2. Symbols denote post-hoc group comparisons of overall effects: Phase 1, **p* < 0.05 FHS vs. GCS controls; Phase III, #*p* < 0.05 FCS-GHS vs. GCS-GCS, †*p* < 0.05 FHS-GHS vs. GCS-GCS, ω*p* < 0.05 FHS-FHS vs. GCS-GCS, δ*p* < 0.05 FHS-GHS vs. FHS-FHS.

**Appendix Table 1. Summary of Multivariate Analysis**

**of Group Differences in Change in Daily Measurements of Blood Pressure and Cumulative Sodium**

|  | **Mean Arterial Blood Pressure** | | |  | **Net Sodium, Adjusted** | | |  | **Cumulative Sodium** | | |
| --- | --- | --- | --- | --- | --- | --- | --- | --- | --- | --- | --- |
|  | **Day** | **Group** | **Day x Group** |  | **Day** | **Group** | **Day x Group** |  | **Day** | **Group** | **Day x Group** |
| **Phase 1** |  |  |  |  |  |  |  |  |  |  |  |
| Week 1 | **31.73,**  **p < 0.001** | 2.80,  p = 0.055 | **2.45,**  **p = 0.002** |  | 0.04,  p = 1.00 | *3.61,*  *p = 0.023* | 1.21,  p = 0.267 |  | **10.02,**  **p < 0.001** | **21.26,**  **p < 0.001** | **2.43,**  **p = 0.002** |
| Week 2 | **6.64,**  **p < 0.001** | 2.78,  p = 0.057 | 1.40,  p = 0.153 |  | 0.015,  p = 1.00 | 2.64,  p = 0.066 | 1.18,  p = 0.299 |  | **28.52,**  **p < 0.001** | **25.77,**  **p < 0.001** | **2.69,**  **p = 0.001** |
| Week 3 | **3.89,**  **p = 0.012** | 0.81,  p = 0.497 | *1.92,*  *p = 0.041* |  | 0.01,  p = 1.00 | **5.56,**  **p = 0.003** | 1.17,  p = 0.319 |  | **15.32,**  **p < 0.001** | **32.62,**  **p < 0.001** | **2.62,**  **p = 0.005** |
|  |  |  |  |  |  |  |  |  |  |  |  |
| **Phase 3** |  |  |  |  |  |  |  |  |  |  |  |
| Week 1 | **6.35,**  **p < 0.001** | **4.10,**  **p = 0.014** | **2.64,**  **p = 0.001** |  | 0.02,  p = 1.00 | **8.45,**  **p < 0.001** | 1.60,  p = 0.076 |  | **16.90,**  **p < 0.001** | **9.96,**  **p < 0.001** | **3.08,**  **p < 0.001** |
| Week 2 | 1.29,  p = 0.296 | *3.09,*  *p = 0.040* | **2.04,**  **p = 0.015** |  | 0.02,  p = 1.00 | **5.27,**  **p = 0.004** | 0.91,  p = 0.566 |  | **28.88,**  **p < 0.001** | **14.61,**  **p < 0.001** | **2.56,**  **p = 0.002** |
| Week 3 | 1.18,  p = 0.340 | **4.30,**  **p = 0.011** | *2.15,*  *p = 0.021* |  | 0.01,  p = 1.00 | **4.74,**  **p = 0.007** | 1.38,  p = 0.19 |  | **27.37,**  **p < 0.001** | **22.71,**  **p < 0.001** | **2.75,**  **p = 0.003** |

*Note:* Statistical results from a multivariate profile analysis of group differences in daily measurements across each week of the study design; all reported values are F-tests with significance testing. All effects in a measurement are estimated in a single statistical model by week of each phase of the design. The effects of interest are the main effect of Group (between-group differences in the weekly average) and the interaction Day × Group (between-group differences in the change during the week). Uncorrected *p*-values are reported; bold effects indicate results that are significant after correction for type I error rate control within each study phase (α’ = 0.02) and italics indicate nominally significant results that did not survive correction. Analysis included 4 experimental groups based on Phase 3 randomized assignment, which provided comparable statistical power across the study design and tested that FHS groups were equivalent prior to dietary change at Phase 3. Net sodium measurements were adjusted for daily calorie intake and urine volume.

**Appendix Table 2. Summary of Multivariate Analysis**

**of Group Differences in Change in Daily Measurements of Unadjusted Net Potassium and Sodium**

|  | **Net Potassium, Unadjusted** | | |  | **Net Sodium, Unadjusted** | | |
| --- | --- | --- | --- | --- | --- | --- | --- |
|  | **Day** | **Group** | **Day x Group** |  | **Day** | **Group** | **Day x Group** |
| **Phase 1** |  |  |  |  |  |  |  |
| Week 1 | 1.96,  p = 0.099 | 0.87,  p = 0.465 | 1.07,  p = 0.391 |  | **13.08,**  **p < 0.001** | **57.70,**  **p < 0.001** | **2.38,**  **p = 0.003** |
| Week 2 | 0.62,  p = 0.713 | 2.45,  p = 0.081 | 1.36,  p = 0.172 |  | **4.47,**  **p = 0.003** | **32.67,**  **p < 0.001** | *1.94,*  *p = 0.022* |
| Week 3 | 2.21,  p = 0.092 | 1.22,  p = 0.318 | 0.77,  p = 0.676 |  | 1.43,  p = 0.247 | **44.44,**  **p < 0.001** | 0.68,  p = 0.767 |
|  |  |  |  |  |  |  |  |
| **Phase 3** |  |  |  |  |  |  |  |
| Week 1 | **9.26,**  **p < 0.001** | 0.30,  p = 0.828 | 1.18,  p = 0.294 |  | **7.09,**  **p < 0.001** | **14.49,**  **p < 0.001** | **3.07,**  **p < 0.001** |
| Week 2 | 1.93,  p = 0.111 | 1.27,  p = 0.301 | 0.90,  p = 0.575 |  | 1.16,  p = 0.356 | **15.04,**  **p < 0.001** | 0.925,  p = 0.552 |
| Week 3 | 0.42,  p = 0.795 | 0.58,  p = 0.631 | 1.72,  p = 0.075 |  | 2.20,  p = 0.093 | **15.90,**  **p < 0.001** | 1.14,  p = 0.335 |

*Note:* Statistical results from a multivariate profile analysis of group differences in daily measurements across each week of the study design; all reported values are F-tests with significance testing. All effects in a measurement are estimated in a single statistical model by week of each phase of the design. The effects of interest are the main effect of Group (between-group differences in the weekly average) and the interaction Day × Group (between-group differences in the change during the week). Uncorrected *p*-values are reported; bold effects indicate results that are significant after correction for type I error rate control within each study phase (α’ = 0.02) and italics indicate nominally significant results that did not survive correction. Analysis included 4 experimental groups based on Phase 3 randomized assignment, which provided comparable statistical power across the study design and tested that FHS groups were equivalent prior to dietary change at Phase 3.
